# Transcriptomic Profiling Identifies Key Genes and ERBB Signaling Pathway Associated with Aggressive Behavior in Muscovy Ducks (*Cairina moschata*)

**DOI:** 10.3390/ani16060951

**Published:** 2026-03-18

**Authors:** Ai Liu, Xuping Wang, Xuan Zhou, Biqiong Yao, Jinjin Zhu, Yifu Rao, Fuyou Liao, Bingnong Yao, Surintorn Boonanuntan, Shenglin Yang

**Affiliations:** 1 Key Laboratory of Animal Genetics, Breeding and Reproduction in the Plateau Mountainous Region, Ministry of Education, Guizhou University, Guiyang 550025, China; liuai4735@163.com (A.L.); 17335774638@163.com (X.W.); 15286056403@163.com (X.Z.); yaobiqiong99@163.com (B.Y.); jinjinz1230@163.com (J.Z.); m18334067492@163.com (Y.R.); liaofuyou1@126.com (F.L.); ybn2226516535@163.com (B.Y.); 2 School of Materials Science and Engineering, Guizhou Minzu University, Guiyang 550025, China; 3 School of Animal Production and Innovation, Suranaree University of Technology, Nakhon Rachasima 30000, Thailand; surinton2012@gmail.com

**Keywords:** Muscovy duck (*Cairina moschata*), aggressive behavior, hypothalamus, transcriptomic profiling, ERBB signaling pathway, evolutionary conservation

## Abstract

Aggressive behavior in Muscovy ducks (Cairna moschata) has become a predominant concern in intensive farming systems, leading to reduced animal welfare and production losses. To unravel the molecular mechanisms underlying this behavior, transcriptomic profiling was performed on the hypothalamus, a key regulatory hub for aggressive responses. A total of 120 healthy 60-day-old female Muscovy ducks were continuously monitored for 24 h/day over one month using Media Recorder 2.0 software. Based on instantaneous and continuous behavioral observations, the ducks were categorized into three groups: aggressor (Experimental group I, actively attacking conspecifics), victim (Experimental group II, receiving aggression), and non-aggressive (Control group, no aggressive interactions). Hypothalamic tissues were collected from each group (n = 4 per group) for Illumina HiSeq 2000 high-throughput transcriptome sequencing. Functional annotation and enrichment analysis of differentially expressed genes (DEGs) were performed using Gene Ontology (GO) and Kyoto Encyclopedia of Genes and Genomes (KEGG) databases, followed by quantitative real-time PCR (qRT-PCR) validation. GO analysis identified 626 DEGs in the aggressor group and 649 DEGs in the victim group compared to the control group, with 26 DEGs directly involved in aggressive behavior regulation. Integration of GO and KEGG annotations revealed 69 candidate genes associated with aggressive behavior, enriched in two GO terms (behavior [GO:0007610] and sensory perception of pain [GO:0019233]) and the ERBB signaling pathway (map04012). qRT-PCR validation of 14 randomly selected candidate genes (e.g., NPY, ERBB4, MAPK9, PRDM12) confirmed that their expression patterns were consistent with transcriptomic data, verifying the reliability of the sequencing results. These findings provide novel insights into the molecular genetic basis of aggressive behavior in Muscovy ducks and lay a foundation for developing targeted strategies to mitigate aggression in intensive farming systems.

## Simple Summary

Aggressive behavior severely impairs animal welfare and causes production losses in intensive Muscovy duck (*Cairina moschata*) farming. To elucidate its molecular mechanisms, we performed hypothalamic transcriptomic profiling of 120 60-day-old female ducks stratified via 24-h continuous monitoring into three groups: aggressor, victim, and control. Illumina HiSeq 2500 sequencing and differential expression analysis identified 626 DEGs in the aggressor group and 649 in the victim group vs. the control group, with 26 overlapping DEGs linked to aggression. Integrative GO and KEGG analyses revealed 69 candidate genes significantly enriched in the behavior (GO:0007610) and sensory perception of pain (GO:0019233) terms, as well as the evolutionarily conserved ERBB signaling pathway (map04012). qRT-PCR validation of 14 key genes (e.g., NPY, ERBB4) confirmed transcriptomic data reliability. These findings provide novel insights into the genetic basis of duck aggression and a framework for targeted mitigation strategies in intensive farming.

## 1. Introduction

The Muscovy duck (*Cairina moschata*), a tropical waterfowl native to Central and South America, is valued in agricultural systems for its strong environmental adaptability, high reproductive performance, and superior meat quality [1]. Unlike most domestic duck breeds derived from the mallard (Anas platyrhynchos), the Muscovy duck represents a distinct evolutionary lineage [2]. Introduced to China centuries ago, it is now predominantly farmed in Fujian, Taiwan, Hubei, Hainan, and Guizhou provinces [3]. With the rapid intensification of Muscovy duck production systems, abnormal behaviors—particularly aggression—have emerged as a critical barrier to sustainable farming [4]. Under high stocking densities and the absence of environmental enrichment (e.g., litter substrates, water baths), ducks frequently exhibit injurious behaviors such as feather pecking and cannibalism, which not only impair animal welfare but also reduce feed conversion efficiency and survival rates [5,6]. Environmental modifications, including provision of water bathing facilities, foraging substrates (e.g., straw or hay), and outdoor access, have been shown to alleviate aggressive tendencies in Muscovy ducks [7]. Specifically, low stocking densities combined with outdoor foraging areas and bathing resources significantly reduce the incidence of feather pecking and interspecific aggression [8,9].

Poultry behavior—including abnormal phenotypes such as feather pecking and aggression—is a complex trait modulated by the interplay of environmental stimuli and genetic determinants. Early hypotheses proposed feather pecking as a redirected foraging behavior, but this theory has not been substantiated by subsequent empirical research [10,11]. Dietary supplementation with feather meal has been demonstrated to alter the gut microbiome composition [12] and reduce feather-pecking behavior in chickens [13]. While compositional differences in gut microbiota have been observed between high- and low-feather-pecking laying hen lines [14,15,16], the causal relationship between gut microbial communities and feather-pecking behavior remains unclear. Nevertheless, this association is biologically plausible, given the well-documented bidirectional crosstalk between the serotonergic system, gut microbiota, and behavioral regulation [17,18]. Central serotonin (5-hydroxytryptamine, 5-HT) modulates dopamine signaling cascades, thereby exerting a pivotal role in the regulation of reward-seeking behaviors and aggression [19].

Comprehensive genomic and transcriptomic approaches are essential for elucidating the molecular regulatory mechanisms underlying feather pecking and aggression, as they enable the identification of key candidate genes and signaling pathways. Transcriptome sequencing (RNA-seq) is a powerful tool for quantifying global gene expression profiles and linking genomic information to phenotypic variation [20]. To date, RNA-seq has been widely applied to characterize unknown transcripts across diverse species. Most transcriptomic studies of poultry behavior have focused on chickens; for example, 423 DEGs associated with feather pecking were identified in the brains of high- versus low-feather-pecking hens [21]. In Muscovy ducks, RNA-seq has been used to investigate the molecular basis of brooding behavior [3], but no systematic studies have explored the genetic underpinnings of aggressive behavior.

The hypothalamus, a core component of the hypothalamic–pituitary–adrenal (HPA) axis, plays a central role in regulating the fight-or-flight response and aggressive behavior in vertebrates [22]. Notably, aggressive behavior is evolutionarily conserved across vertebrates, and studies in mammals (e.g., mice, silver foxes) have identified key regulatory genes and pathways, but the molecular mechanisms underlying avian aggression remain poorly understood, especially in non-model agricultural species like Muscovy ducks. In the present study, we employed RNA-seq to profile the hypothalamic transcriptome of Muscovy ducks with distinct aggressive phenotypes (aggressive, attacked, and non-aggressive) and validated key DEGs via qRT-PCR. Our objectives were to: (1) uncover the genetic basis of aggressive behavior in Muscovy ducks; (2) explore its molecular regulatory mechanisms, with a focus on evolutionary conservation of key pathways across vertebrates; (3) provide targeted candidate genes/pathways for improving animal welfare and production efficiency in intensive farming systems. This work fills the gap in avian aggression research and contributes to the cross-species understanding of behavior regulatory networks, aligning with the core focus of Genes, Brain and Behavior.

## 2. Materials and Methods

### 2.1. Animals, Housing and Behavioral Monitoring

A total of 120 healthy 60-day-old female Muscovy ducks with similar body weight and consistent genetic background were purchased from Brother Duck Farm in Sansui County, Guizhou Province, China. The behavior was recorded and analyzed 24 h a day for 1 month using The Medial Recorder 2.0 software (Noldus Information Technology bv, Wageningen, The Netherlands). The instantaneous observation method and continuous observation method were adopted to screen the ducks into three groups. Each group was reared in two replicate cages (3 × 3 × 0.7 m) with corresponding duck numbers per cage, and the three groups were divided as follows: Control Group: Ducks with no aggressive behavior and normal performance. Experiment Group I: Ducks showing aggressive behavior. Experiment Group II: Ducks showing attacked behavior. All groups were reared under standardized conditions: 12:12 h light-dark cycle, temperature 22 ± 2 °C, and relative humidity 60% ± 5%. They were fed a commercial corn-soybean meal diet three times daily (07:00–08:00, 11:30–12:30, 17:30–18:30) with ad libitum access to clean drinking water. The housing environment was regularly cleaned, disinfected, and mechanically ventilated to ensure hygiene and air quality. 

For 24-h continuous behavioral monitoring, three high-definition video cameras were mounted at different angles on the inner roof of each cage and connected to Media Recorder 2.0 software. At the end of the experiment, hypothalamus samples were collected from four randomly selected ducks per group for transcriptomic profiling.

### 2.2. Hypothalamus Sample Collection

Following the behavioral monitoring period, four randomly selected ducks per group were anesthetized with intravenous sodium pentobarbital (100 mg/kg body weight) and euthanized by exsanguination in accordance with the Guidelines for the Ethical Treatment of Experimental Animals. Hypothalamic tissues were rapidly dissected from four biological replicates per group, immediately snap-frozen in liquid nitrogen after diethyl pyrocarbonate (DEPC, Beijing Labgic Technology Co., Ltd., Beijing, China) treatment to inhibit RNA degradation, and stored at −80 °C until RNA extraction. A subset of samples was transported on dry ice to Beijing Nuohe Zhiyuan Technology Co., Ltd. (Beijing, China) for cDNA library construction and high-throughput sequencing.

### 2.3. RNA Isolation, cDNA Library Preparation and Transcriptome Sequencing

Total RNA was extracted from hypothalamic tissues using the TransZol Up Plus RNA Kit (Invitrogen, Carlsbad, CA, USA) following the manufacturer’s protocol. RNA quality was assessed by three methods: (1) 1% agarose gel electrophoresis for degradation and contamination detection; (2) NanoPhotometer^®^ spectrophotometer (IMPLEN, Munich, Germany) for purity (A260/A280 ratio: 1.8–2.0); (3) Bioanalyzer 2100 system (Agilent Technologies, Santa Clara, CA, USA) for integrity (RNA Integrity Number, RIN ≥ 7.0). Only high-quality RNA samples (RIN ≥ 7.0) were used for subsequent experiments [23,24], and equal amounts of total RNA from three technical replicates per group were pooled to minimize individual variation.

cDNA libraries were constructed using the NEBNext^®^ Ultra™ RNA Library Prep Kit for Illumina^®^ (New England Biolabs, Ipswich, MA, USA) with index codes for sample distinction. Briefly, mRNA was enriched from total RNA using poly-T oligo-attached magnetic beads, fragmented in NEBNext First Strand Synthesis Reaction Buffer (5×) at 94 °C for 8 min, and reverse-transcribed into first-strand cDNA with random hexamer primers and M-MuLV Reverse Transcriptase (RNase H^−^). Second-strand cDNA was synthesized using DNA Polymerase I and RNase H, with overhangs converted to blunt ends via exonuclease/polymerase activities. After 3’ end adenylation, NEBNext Adaptors with hairpin loop structures were ligated, and 150–200 bp library fragments were purified using the AMPure XP system (Beckman Coulter, Brea, CA, USA) [25]. USER Enzyme (New England Biolabs, Ipswich, MA, USA) digested adaptor sequences at 37 °C for 15 min, followed by heat inactivation at 95 °C for 5 min. PCR amplification was performed with Phusion High-Fidelity DNA polymerase, Universal PCR primers, and Index (X) Primer, and final products were purified using the AMPure XP system. Library quality was assessed on the Agilent Bioanalyzer 2100 system [26]. Clustering of index-coded samples was conducted on a cBot Cluster Generation System using the TruSeq PE Cluster Kit v3-cBot-HS (Illumina, San Diego, CA, USA), and sequencing was performed on an Illumina HiSeq 2500 platform to generate 125 bp paired-end reads.

Clustering of index-coded samples was performed on a cBot Cluster Generation System using the TruSeq PE Cluster Kit v3-cBot-HS (Illumina, San Diego, CA, USA) according to the manufacturer’s instructions. Library sequencing was conducted on an Illumina HiSeq 2500 platform, generating 125 bp paired-end reads.

### 2.4. Transcriptome Data Analysis

Raw fastq reads were filtered using in-house Perl scripts to remove adapters, poly-N reads (N content >5%), and low-quality reads (Q-value <20). Clean reads with Q20 were retained, and quality metrics (Q20, Q30, GC content) were calculated for reliability verification. Clean reads were mapped to the Muscovy duck reference transcriptome (GenBank accession: GGZN00000000) using Bowtie 2.2.3, and paired-end clean reads were aligned to the reference genome with TopHat v2.0.12, a splice junction-aware aligner optimized for eukaryotic transcriptomes.

HTSeq v0.6.1 was used to count reads mapped to each gene, and fragments per kilobase of transcript per million mapped reads (FPKM) was calculated for gene expression level estimation. Differential expression analysis between groups was performed using the DESeq R package (v1.18.0), which employs a negative binomial distribution model for RNA-seq data. *p*-values were adjusted via the Benjamini–Hochberg method to control the false discovery rate (FDR), and genes with adjusted *p*-value <0.05 were defined as significantly differentially expressed.

GO enrichment analysis of differentially expressed genes (DEGs) was performed using the GOseq R package (v1.16.2) to correct gene length bias. GO terms were categorized into biological process (BP), cellular component (CC), and molecular function (MF), with terms having corrected *p*-value <0.05 considered significantly enriched. KEGG pathway enrichment analysis was conducted using KOBAS v3.0 software to identify associated signaling pathways. Functional annotation was integrated via GO and KEGG databases for comprehensive characterization of DEG biological functions [27], and data were visualized using the ggplot2 R package and Microsoft Excel 2019 (Redmond, WA, USA).

### 2.5. Quantitative Real-Time PCR (qRT-PCR) Validation

Fourteen aggression-related DEGs were randomly selected for qRT-PCR validation (Table 1). Primers were designed using Primer 5.0 software (Premier Biosoft, Palo Alto, CA, USA) based on transcriptome results, with GAPDH as the reference gene. cDNA was synthesized from 1 μg total RNA using the PrimeScript™ RT Reagent Kit with gDNA Eraser (TaKaRa, Dalian, China) to eliminate genomic DNA contamination. qRT-PCR was performed on a StepOnePlus™ Real-Time PCR System (Applied Biosystems, Foster City, CA, USA) using TB Green^®^ Premix Ex Taq™ II (TaKaRa, Dalian, China) in a 20 μL reaction system (10 μL TB Green^®^ Premix Ex Taq™ II, 0.4 μL each forward/reverse primer (10 μM), 2 μL cDNA template, 7.2 μL nuclease-free water). Cycling conditions were initial denaturation at 95 °C for 30 s, followed by 40 cycles of 95 °C for 5 s and primer-specific annealing/extension for 30 s (Table 1). Melting curve analysis (60–95 °C) confirmed primer specificity. Each sample was analyzed in triplicate, and relative gene expression levels were calculated using the 2^−^ΔΔCt method [28], where ΔCt = Ct(target gene) − Ct(reference gene), and ΔΔCt = ΔCt(experimental group) − ΔCt(control group), representing the relative fold change in gene expression between the experimental and control groups.

## 3. Results

### 3.1. Quality Assessment of Transcriptomic Data

Transcriptomic sequencing of hypothalamic tissues from three phenotypic groups (aggressor, victim, and control Muscovy ducks) yielded high-quality sequencing data, as evidenced by rigorous quality control metrics (Table 2). All RNA samples exhibited RNA Integrity Number (RIN) values ≥7.0, indicating minimal degradation and suitability for library construction. Sequencing error rates were consistently low (0.02%) across all samples, with Q20 and Q30 values exceeding 94.8% and 88.05%, respectively. The GC content ranged from 45.37% to 49.97%, which is consistent with the genomic characteristics of avian species and further confirms the reliability of the sequencing data [24]. These metrics collectively demonstrate that the transcriptomic dataset is of sufficient quality for subsequent differential expression and functional enrichment analyses.

### 3.2. Genome Mapping and Read Distribution

**Figure 1:**
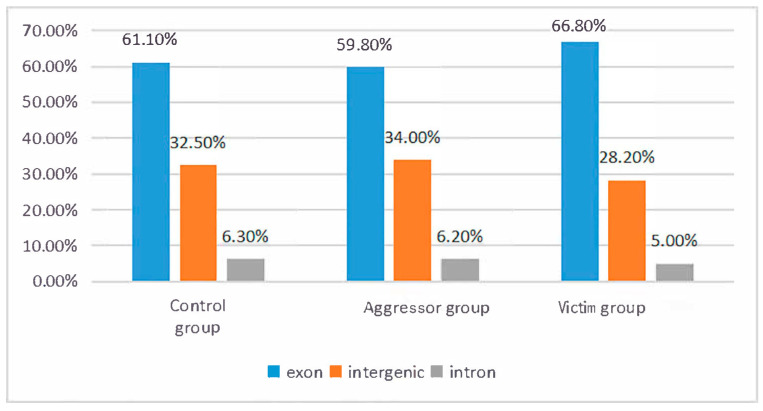
Proportion of reads in exonic, intronic and intergenic regions.

Clean reads were aligned to the Muscovy duck reference genome (GenBank accession: GGZN00000000) using TopHat v2.0.12. Mapping efficiency varied slightly among groups: the control group exhibited the highest overall mapping rate (72.24%), followed by the aggressor group (71.24%), while the victim group showed a relatively lower rate (64.58%) (Table 3). The reduced mapping efficiency in the victim group is likely attributable to incomplete annotation of the Muscovy duck reference genome, a common challenge in transcriptomic studies of non-model species [29]. Nevertheless, all groups achieved unique mapping rates >63%, and read distribution analysis revealed that >85% of clean reads mapped to exonic regions, with the remainder distributed in intronic or intergenic regions (Figure 1). As shown in Figure 1 (Distribution of clean reads mapped to different genomic regions), the high proportion of exonic reads confirms that the sequencing data effectively captured coding transcript information, which is critical for ensuring the validity of downstream gene expression analyses.

### 3.3. Identification of Differentially Expressed Genes (DEGs)

**Figure 2:**
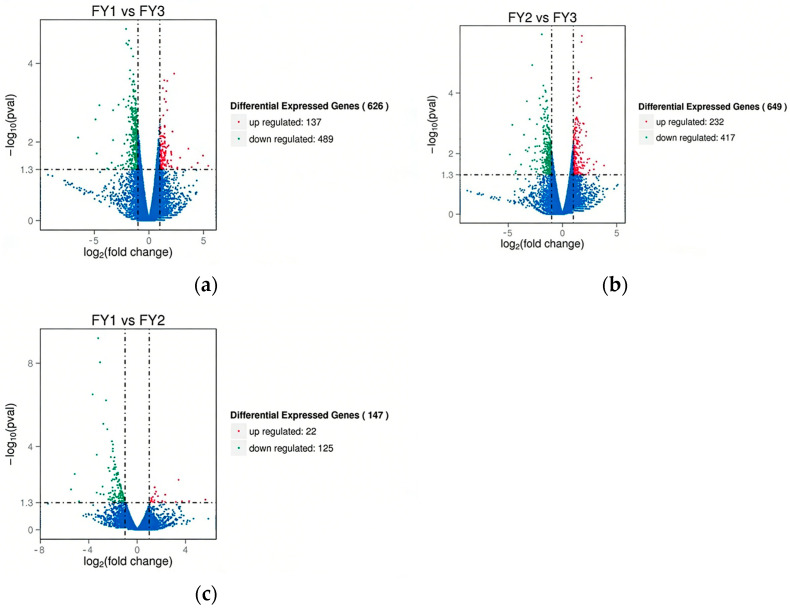
(**a**) Volcano plots of differentially expressed genes between aggressor group (FY1) and control group(FY3). (**b**) Volcano plots of DEGs between victim group (FY2) and control group (FY3). (**c**) Volcano plots of DEGs between aggressor group (FY1) and victim group (FY2). The red color represents up regulated genes, the green color denotes down regulated genes while the blue means non-expression genes. The horizontal dashed line indicates the threshold for statistical significance (*p* < 0.05). The vertical dashed lines represent the threshold for a 2-fold change in expression. Genes above the horizontal line and outside the vertical lines were considered differentially expressed.

Differential expression analysis between groups was performed using the DESeq R package (v1.18.0) with a false discovery rate (FDR) <0.05 as the threshold for significance. The distribution of DEGs in each pairwise comparison is visualized in the volcano plots (Figure 2). Specifically, Figure 2a shows DEGs between the aggressor group (FY1) and the control group (FY3), Figure 2b shows DEGs between the victim group (FY2) and the control group (FY3), and Figure 2c shows DEGs between the aggressor group (FY1) and the victim group (FY2). In these volcano plots, the x-axis represents log_2_ (fold change) and the y-axis represents −log_10_ (adjusted *p*-value), with red dots indicating significantly up-regulated genes, green dots indicating significantly down-regulated genes, and blue dots indicating non-differentially expressed genes. Compared with the control group, the aggressor group harbored 626 DEGs, including 137 up-regulated and 489 down-regulated genes. The victim group exhibited 649 DEGs relative to the control group, with 203 up-regulated and 446 down-regulated transcripts.

**Figure 3:**
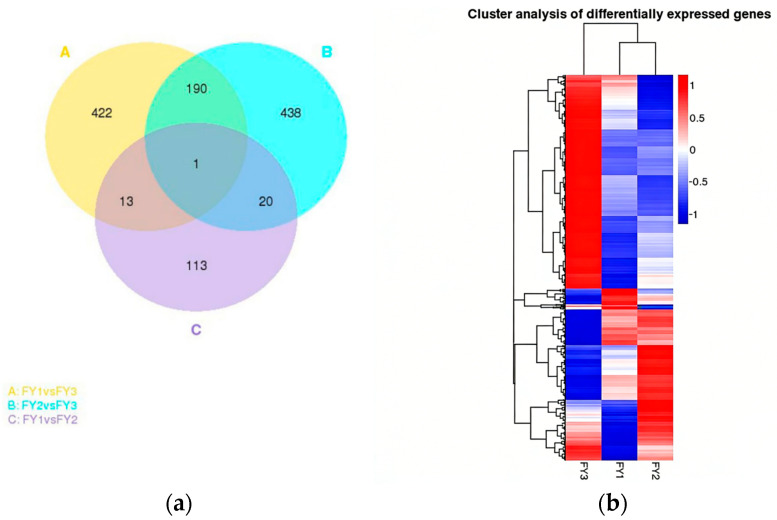
(**a**) Venn diagram of differentially expressed genes. (**b**) Cluster analysis of differentially expressed genes. The overall FPKM hierarchical clustering map was obtained with log10 (FPKM + 1) for normalization. Different columns represent groups, different rows represent genes, different colors represent expression levels (red: highly expressed genes, blue: lowly expressed genes).

Venn diagram analysis further identified 26 DEGs commonly shared between the aggressor and victim groups (Figure 3a). Figure 3 also includes a hierarchical clustering heatmap of DEGs across all groups (Figure 3b), where FPKM values were normalized by log_10_(FPKM + 1); columns represent experimental groups, rows represent DEGs, and the color gradient (red = high expression, blue = low expression) clearly distinguishes the expression patterns of DEGs among the three groups. The 26 shared DEGs are directly implicated in the regulation of aggressive behavior—consistent with their potential role as core molecular mediators of social interaction-related phenotypes.

### 3.4. GO and KEGG Enrichment Analysis 

**Figure 4:**
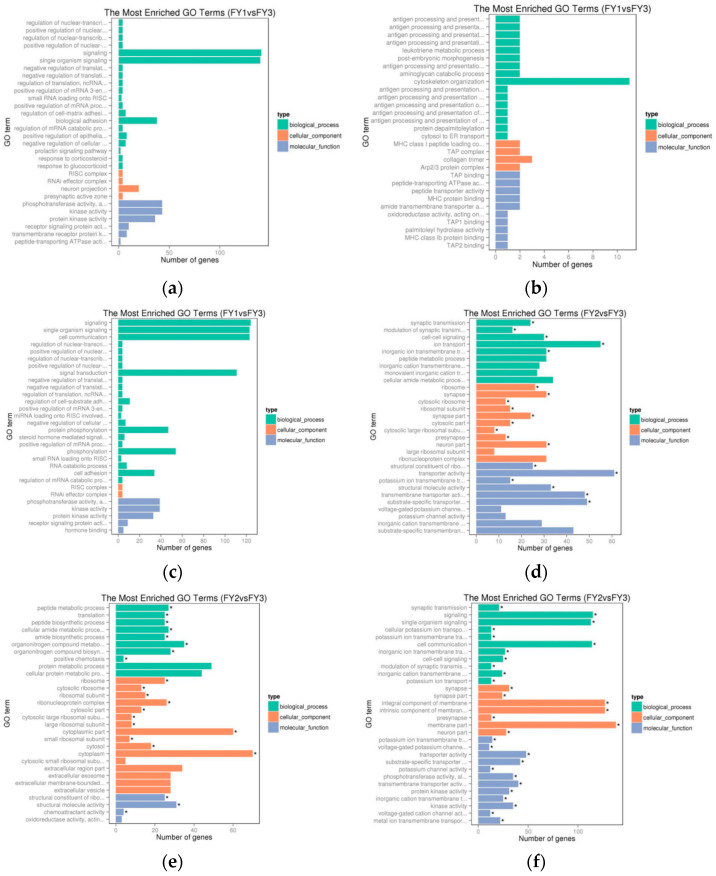
Bar graphs of differentially expressed genes of aggressor and victim groups in GO analysis. (**a**–**c**) All expression, up-regulated expression and down-regulated expression in aggressor group. (**d**–**f**) All expression, up-regulated expression and down-regulated expression in victim group. Asterisks (*) indicate significantly enriched GO terms (adjusted *p*-value < 0.05).

Functional annotation of DEGs was conducted to elucidate biological processes and signaling pathways associated with aggressive behavior. GO enrichment analysis (corrected *p*-value < 0.05) revealed that the 69 candidate genes linked to aggression were significantly enriched in two key GO terms: behavior (GO:0007610) and sensory perception of pain (GO:0019233). The results of the GO enrichment analysis are presented as bar graphs in Figure 4, which stratifies DEGs into all, upregulated, and downregulated subsets for both the aggressor and victim groups. Specifically, Figure 4a–c show the enriched GO terms for all, up-regulated, and down-regulated DEGs in the aggressor group, respectively, while Figure 4d–f show the corresponding results for the victim group. In these bar graphs, the x-axis represents the number of DEGs enriched in each GO term, and the y-axis represents the top enriched GO terms in the biological process category. Differentially expressed genes involved in the above biological processes and metabolic pathways were collated and analyzed. Candidate genes related to aggressive behavior are listed in Table 4. The behavior term encompasses genes involved in social interaction, aggression, and stress response, while sensory perception of pain suggests a potential link between nociceptive signaling and aggressive behavior—consistent with studies in chickens showing that pain perception modulates injurious pecking [28,29].

**Figure 5:**
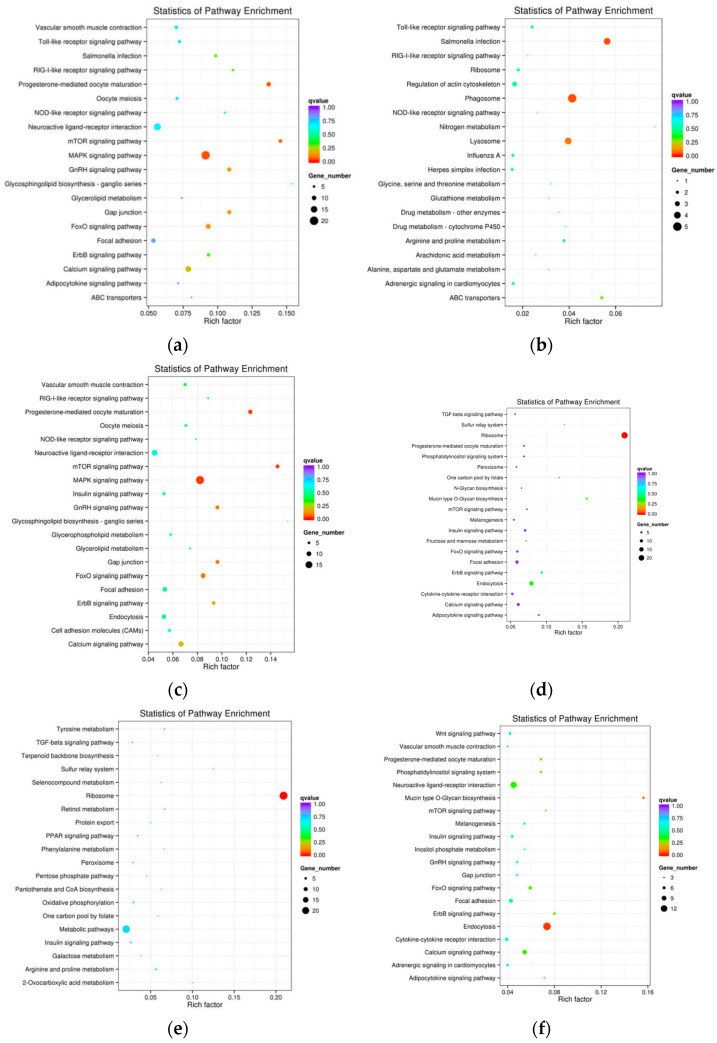
Scatter diagrams of differentially expressed genes of aggressor and victim groups in KEGG analysis. (**a**–**c**) All expression, up-regulated expression and down-regulated expression in aggressor group. (**d**–**f**) All expression, up-regulated expression and down-regulated expression in victim group.

KEGG pathway enrichment analysis further identified the ERBB signaling pathway (map04012) as the most significantly enriched pathway (*p* < 0.01). The results of the KEGG enrichment analysis are presented as scatter plots in Figure 5, which also stratifies DEGs into all, up-regulated, and down-regulated subsets for both groups (Figure 5a–c for the aggressor group, Figure 5d–f for the victim group). In these scatter plots, the x-axis represents the enrichment factor (ratio of DEGs to total genes in the pathway), the y-axis represents the pathway name, dot size indicates the number of DEGs in the pathway, and dot color indicates the corrected *p*-value. The ERBB family of receptor tyrosine kinases (including ERBB4, a candidate gene validated in this study) plays critical roles in neural development, synaptic plasticity, and behavioral regulation [30,31]. Dysregulation of ERBB signaling has been associated with aggressive behavior in mammals: for example, ERBB4 knockout mice exhibit increased aggressive tendencies [32], and dysregulated Neuregulin-1/ErbB signaling is observed in the prefrontal cortex of stress-induced aggressive rats. Our observation of ERBB4 downregulation in aggressive Muscovy ducks mirrors these mammalian findings, highlighting the evolutionary conservation of the ERBB signaling pathway in behavioral modulation across vertebrates. This cross-species consistency strengthens the biological significance of our findings, a key focus of Genes, Brain and Behavior.

The differentially expressed genes shared by the two experimental groups were annotated to two GO terms, namely behavior and sensory perception of pain, while the ERBB signaling pathway was identified in the KEGG pathway database. A total of 74 enriched differentially expressed genes were screened as key candidate genes related to the aggressive behavior of Cairna moschata (Table 5).

### 3.5. qRT-PCR Validation of Candidate Genes

**Figure 6:**
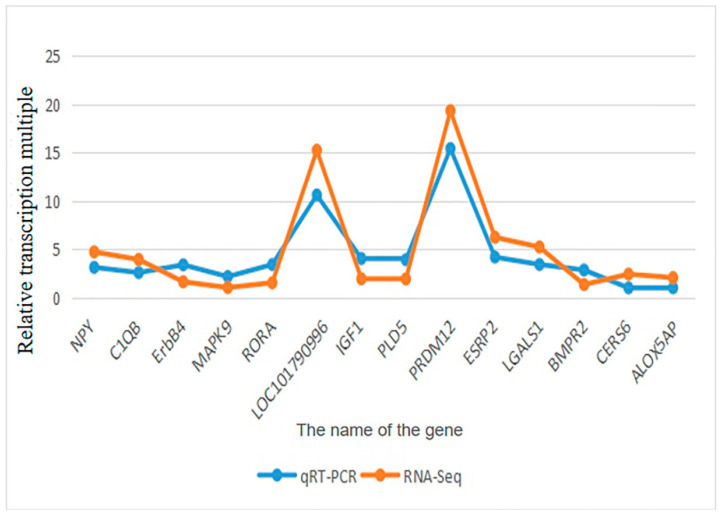
Comparison of the relative transcriptional multiples in fourteen genes by qRT-PCR and RNA-Seq.

To validate the reliability of the transcriptomic data, 14 candidate genes (including NPY, ERBB4, MAPK9, and PRDM12) were randomly selected for qRT-PCR analysis, with GAPDH as the reference gene (Table 1). The correlation between the relative expression levels determined by RNA-seq and qRT-PCR is shown in Figure 6 (Validation of DEG expression levels by qRT-PCR and RNA-seq). In this figure, the x-axis represents the log_2_ (fold change) from RNA-seq, the y-axis represents the log_2_ (fold change) from qRT-PCR, and the regression line with the coefficient of determination (R^2^ = 0.89) indicates a strong positive correlation between the two methods.

For example, NPY (neuropeptide Y), a well-characterized regulator of aggression and stress response in poultry [33], was significantly up-regulated in the aggressor group (fold change = 2.34, FDR = 0.02) in RNA-seq data, and this up-regulation was confirmed by qRT-PCR (fold change = 2.28, *p* = 0.03). Similarly, ERBB4, a core component of the ERBB signaling pathway, exhibited down-regulation in both aggressor and victim groups relative to controls, with qRT-PCR results mirroring the transcriptomic profile. The strong correlation between RNA-seq and qRT-PCR data confirms the accuracy and reproducibility of the sequencing results.

## 4. Discussion

Aggressive behavior in intensive Muscovy duck farming compromises animal welfare and reduces production efficiency, making it a critical constraint to sustainable agriculture [4,5,6]. The hypothalamus, as a central regulator of the HPA axis and social behavior, is an ideal target for investigating the molecular basis of aggression [23,34]. In this study, we employed RNA-seq to identify DEGs and signaling pathways associated with aggressive phenotypes in Muscovy ducks, providing novel insights into the genetic mechanisms underlying this complex trait.

The identification of 626 and 649 DEGs in the aggressor and victim groups, respectively, relative to controls (Figure 2), reflects the transcriptional complexity of aggressive behavior. The 26 shared DEGs between the two experimental groups (Figure 3a) likely represent core regulators of social interaction—consistent with the notion that both initiating and receiving aggression involve overlapping molecular pathways [34]. For instance, PRDM12 (PR domain zinc finger protein 12), a shared DEG, has been linked to pain perception and neural development in mammals [35], and its dysregulation in our study suggests a potential role in mediating the sensory and behavioral responses to aggression in ducks. The hierarchical clustering heatmap (Figure 3b) further confirms the distinct expression patterns of DEGs among the three groups, providing additional evidence for the transcriptional differences associated with aggressive phenotypes.

GO enrichment of the behavior term (Figure 4) confirms that the candidate genes are functionally relevant to aggressive phenotypes. The enrichment of sensory perception of pain is particularly noteworthy, as it supports the hypothesis that aggressive behavior in poultry may be modulated by nociceptive signaling [30]. Previous studies have shown that pain-induced stress can exacerbate aggressive tendencies in chickens [29], and our findings extend this observation to Muscovy ducks, highlighting the need to consider pain management as part of strategies to mitigate aggression in intensive farming. The bar graphs in Figure 4 clearly show the distribution of DEGs in these key GO terms across different groups and expression subsets, reinforcing the functional relevance of these genes to aggressive behavior.

The enrichment of the ERBB signaling pathway (Figure 5) is a key novel finding of this study, and its evolutionary conservation across vertebrates enhances the translational value of our work—an important criterion for Genes, Brain and Behavior. The ERBB pathway, primarily known for its roles in cell proliferation and development, has recently emerged as a regulator of social behavior [30,31]. ERBB4, a member of the ERBB receptor family and a candidate gene in our study, is highly expressed in the hypothalamus and modulates synaptic function and neurotransmitter release. In mice, ERBB4 knockout leads to increased aggression [36], and dysregulated ERBB signaling is associated with stress-induced aggressive phenotypes in rats. Our observation of ERBB4 downregulation in aggressive Muscovy ducks not only confirms the conserved role of this pathway in avian aggression but also provides a cross-species validation of ERBB signaling as a core regulator of aggressive behavior. Notably, a recent study in silver foxes (a classic model for aggressive behavior) identified hypothalamic gene expression differences related to neural signaling pathways, and our findings on ERBB signaling further expand the conserved behavioral regulatory network across mammals and avians. The scatter plots in Figure 5 highlight the significance of the ERBB signaling pathway by showing its high enrichment factor and low corrected *p*-value, further supporting its role in regulating aggressive behavior in Muscovy ducks. This finding opens new avenues for investigating the molecular crosstalk between neural development and social behavior in ducks, and contributes to the broader understanding of evolutionarily conserved behavior regulatory mechanisms.

NPY, a neuropeptide with well-documented roles in appetite regulation and stress response, was significantly upregulated in the aggressive group. Consistent with our results (Figure 6), NPY overexpression has been associated with increased aggression in chickens [37] and mammals [38], likely through modulation of the HPA axis and serotonergic signaling. The upregulation of NPY in aggressive ducks may reflect a compensatory response to stress induced by repeated aggressive interactions, highlighting the integration of metabolic and behavioral regulation in response to social stress. The strong correlation between RNA-seq and qRT-PCR data (Figure 6) ensures the reliability of this finding, providing a solid foundation for future functional studies on NPY in Muscovy duck aggression.

This study has several limitations that should be acknowledged, and addressing these in future work will further enhance the impact of our findings—consistent with the expectations of Genes, Brain and Behavior for rigorous scientific discussion. First, the Muscovy duck reference genome is incompletely annotated, which may have contributed to the lower mapping rate in the attacked group and potential underrepresentation of DEGs; future studies could integrate de novo assembly to complement the reference genome. Second, the study focused solely on the hypothalamus, but aggressive behavior is regulated by a neural network involving multiple brain regions (e.g., amygdala, prefrontal cortex homologs in birds, such as the nidopallium caudolaterale) [39]; exploring gene expression profiles in these regions will provide a more comprehensive understanding of the neural regulatory network of aggression. Third, the causal relationship between the identified DEGs and aggressive behavior remains to be validated through functional studies (e.g., gene overexpression, knockout, or RNA interference in ducks), which is essential to confirm the regulatory role of candidate genes (e.g., ERBB4, NPY). ErbB signaling has been reported to function in neural development and cell differentiation independently of cell number [40]. Fourth, we did not measure neuroendocrine indicators (e.g., cortisol, serotonin) that are closely linked to aggression and HPA axis activity; integrating transcriptomic data with neuroendocrine profiling will strengthen the mechanistic link between gene expression and behavioral phenotypes. Finally, our behavioral classification was based on observational data; incorporating physiological markers of stress (e.g., blood glucose, corticosterone) could improve the accuracy of phenotypic stratification.

Despite these limitations, our findings provide the first systematic characterization of the hypothalamic transcriptome associated with aggressive behavior in female Muscovy ducks. The identification of candidate genes (e.g., NPY, ERBB4, PRDM12) and the ERBB signaling pathway offers novel targets for developing strategies to mitigate aggression in intensive farming—such as marker-assisted selection for low-aggression phenotypes or targeted pharmacological interventions. Additionally, these results contribute to our understanding of the evolutionary conservation of behavioral regulatory mechanisms across avian and mammalian species.

Collectively, this study delineates key molecular mediators of aggressive behavior in female Muscovy ducks and underscores the value of transcriptomics in deciphering complex behavioral traits in non-model agricultural species. Future research should prioritize validating the functional roles of candidate genes (e.g., NPY, ERBB4) via in vivo and in vitro experiments, while also exploring the interactive effects of genetic factors and environmental variables (e.g., stocking density, foraging substrates) on aggressive phenotypes. Such endeavors will lay a solid foundation for developing science-based strategies to mitigate aggression, thereby advancing animal welfare and promoting sustainable development in the Muscovy duck farming industry.

It should be noted that the results of this study are based solely on female Muscovy ducks. Since male ducks generally exhibit more prominent aggressive behavior in poultry, the findings may not be directly generalized to male individuals. Future studies should include both male and female Muscovy ducks to further verify the molecular mechanisms of aggressive behavior and improve the universality of the research results. The present results reflect correlative relationships, and that further functional validation experiments will be required to verify the exact regulatory roles and molecular mechanisms of these genes and pathways in aggressive behavior.

## 5. Conclusions

In this study, high-throughput transcriptome sequencing (RNA-seq) was employed to systematically screen for genes associated with aggressive behavior in female Muscovy ducks (*Cairina moschata*), using hypothalamic tissues from three phenotypic groups (aggressive, attacked, and non-aggressive). A total of 69 key candidate genes were preliminarily identified, with 26 DEGs commonly shared between the aggressor and victim groups (Figure 3a), suggesting their core regulatory roles in social interaction-related phenotypes. Functional enrichment analysis revealed that these candidate genes were significantly enriched in two critical GO terms—behavior (GO:0007610) and sensory perception of pain (GO:0019233) (Figure 4)—and the ERBB signaling pathway (map04012) (Figure 5). Notably, the ERBB signaling pathway, previously implicated in mammalian behavioral regulation, was confirmed to be involved in avian aggressive behavior, highlighting the evolutionary conservation of this regulatory mechanism.

Furthermore, qRT-PCR validation of 14 candidate genes (including NPY, ERBB4, and PRDM12) confirmed the reliability of the transcriptomic data, with a strong correlation (R^2^ = 0.89) between RNA-seq and qRT-PCR results (Figure 6). These findings not only expand our understanding of the molecular genetic basis of aggressive behavior in female Muscovy ducks but also provide critical foundational data for exploring the molecular regulatory networks underlying this complex trait. The identified candidate genes and ERBB signaling pathway offer novel targets for developing practical strategies to mitigate aggression in intensive Muscovy duck farming, such as marker-assisted selection or targeted nutritional interventions, thereby promoting animal welfare and sustainable production.

## Figures and Tables

**Table 1:** Sequences of primers, annealing temperature and predicted length of target DNA fragment.

Gene	Full Name	Nucleotide Sequence (5′→3′)	Tm (°C)	Length (bp)	Accession No.
NPY	Neturopetide Y	F: CTCTGAGGCACTACATCAACC	48	135	NW_004677092.1
		R: ACCACATCGAAGGGTCTTCAA	47.8		
C1QB	Complement C1q B chain	F: GCCTCAGAATGAAAAA	58	202	NW_004676672.1
		R: AGGAAGCCAGAAAAGA	58		
ErbB4	Erb-b2 receptor tyrosine kinase 4	F: CTTGTGTTCGTGCGTG	58	244	NW_004677206.1
		R: ATAGCGGCGATTGTGT	58		
MAPK9	Mitogen-activated protein kinase 9	F:GTACAGACCTCTTCCC	56	258	NW_004677772.1
		R: GCTTGGCTGGTTTTTA	56		
RORA	RAR related orphan receptor A	F:TTGCTGTGGGGATGTC	57	176	NW_004678221.1
		R:CCCGTTGGTGGTGATA	57		
LOC101790996	Unknown functional gene	F: AAATCTTGATCCTCTC	59.5	104	NW_004677172.1
		R: AAAAATCCTCTTGTTG	59.5		
IGF1	Insulin like growth factor 1	F: CACTGTGTGGTGCTGA	59	190	NW_004677293.1
		R:GGTGGCTTTATTGGAG	59		
PLD5	Phospholipase D family member 5	F: TGGGACAGATGAAGGA	60.5	232	NW_004677435.1
		R: TTTGGGCAGAAGAGTT	60.5		
PRDM12	PR/SET domain 12	F: GGGCTGGAAGAGGAG	60	207	NW_004676566.1
		R: GGAGGACTGGCTGAA	60		
ESRP2	Epithelial splicing regulatory protein 2	F: CAGGAGGATGTGTTGG	58.5	278	NW_004677744.1
		R: GGAATGATGGGAGTTG	58.5		
LGALS1	Galectin 1	F: GATTCCACCCAACGCC	59.5	144	NW_004676738.1
		R: GCACCCCACTCCTCCA	59.5		
BMPR2	Bone morphogenetic protein receptor type 2	F: GTACAGACCTCTTCCC	60	272	NW_004676788.1
		R: TCTTTCCCAAATCATC	60		
CERS6	Ceramide synthase 6	F: TTCCTGCCTTGTGGGTC	58	213	NW_004676960.1
		R:TGTGCGGGGTCTTTGAG	58		
ALOX5AP	Arachidonate 5-lipoxygenase activating protein	F: GTCTACACTGCCAACCA	59	217	NW_004676888.1
		R:TGAGGAACAGGAACAAA	59		
GAPDH	Glyceraldehyde-3-phosphate dehydrogenase	F:ATCTTTCTTGGGTATGGAGTC	58	185	NW_004677060
		R: TTTCATCGTGCTGGGTG	58		

**Table 2:** Quality of the output of the sequenced data.

Group	Sample Name	Raw Reads	Clean Reads	Clean Bases	Error Rate (%)	Q20	Q30	GC Content
Control group	F_2	58,376,644	57,519,912	8.63 G	0.02	96.22	90.62	46.6
	F_3	50,773,564	49,752,982	7.46 G	0.02	95.32	88.64	46.8
	F_4	49,791,448	48,848,604	7.33 G	0.02	95.51	88.98	45.37
Aggressor group	F2	51,109,532	50,041,418	7.51 G	0.02	95.41	89.07	47.51
	F5	48,714,368	47,774,096	7.17 G	0.02	95.65	89.41	46.51
	F6	55,286,690	54,108,252	8.12 G	0.02	95.41	88.95	47.12
Victim group	F_5	48,933,536	47,761,296	7.16 G	0.02	94.8	88.05	49.97
	F_6	55,758,322	54,485,628	8.17 G	0.02	95.05	88.22	46.49
	F_7	55,679,956	53,450,590	8.02 G	0.02	95.72	89.67	47.97

Q20 and Q30 represent the percentage of the Phed of bases which is greater than 20 and 30, respectively.

**Table 3:** Results of the sequenced data, mapping.

Group	Total Mapped	Mapped Rate /%	Uniquely Mapped	Uniquely Mapped Rate/%	Reads Map to ‘+’	Reads Map to ‘+’ Rate	Reads Map to ‘−’	Reads Map to ‘−’ Rate
Control group	35,287,050	72.24	34,840,895	71.32%	17,380,002	35.58%	17,460,893	35.74%
Aggressor group	34,033,433	71.24	33,614,280	70.36%	16,769,863	35.1%	16,844,417	35.26%
Victim group	35,187,890	64.58	34,805,022	63.88%	17,349,206	31.84%	17,455,816	32.04%

**Table 4:** Differential expression statistics of genes related to aggressive behavior.

Group	Related GO Entries	KEGG Pathway ID	The Total Number of Genes Involved
Aggressor group	407	10	558
Victim group	567	4	524

**Table 5:** Key DEGs related to aggressive behavior.

Group	Key Candidate Gene	Gene Description	Accession No.
Aggressor group 43	ELK4	ETS transcription factor	NW_004676641.1
	LOC101802116	Mitogen-activated protein kinase 13	NW_004677425.1
	LOC101793653	Glutamate dehydrogenase 1	NW_004676880.1
	GAD1	Glutamate decarboxylase 1	NW_004676960.1
	DGKQ	Diacylglycerol kinase theta	NW_004685070.1
	DGKE	Diacylglycerol kinase epsilon	NW_004676503.1
	MBOAT2	Membrane bound O-acyltransferase domain containing 2	NW_004676614.1
	PRLR	Prolactin receptor	NW_004676810.1
	IFFO2	Intermediate filament family orphan 2	NW_004676672.1
	PRKCA	Protein kinase C alpha	NW_004676747.1
	PAPSS2	3′-phosphoadenosine 5′-phosphosulfate synthase 2	NW_004676822.1
	PRKACB	Protein kinase cAMP-activated catalytic subunit beta	NW_004677257.1
	BRAF	B-Raf proto-oncogene	NW_004677175.1
	LOC101804248	Unknown	
	TNR	Tenascin R	NW_004676988.1
	ITGA9	Integrin subunit alpha 9	NW_004676605.1
	DPYSL3	Dihydropyrimidinase like 3	NW_004677279.1
	LOC101799402	Afadin	NW_004679826.1
	RPS6KA6	ribosomal protein S6 kinase A6	NW_004676464.1
	IGF1	Insulin-like growth factor 1	NW_004677293.1
	NR3C1	Nuclear receptor subfamily 3 Group C member 1	NW_004677279.1
	NR2C2	Nuclear receptor subfamily 2 group C member 2	NW_004676494.1
	ESRRG	Estrogen related receptor gamma	NW_004676345.1
	LOC101793863	Unknown	
	MAPK10	Mitogen-activated protein kinase 10	NW_004676534.1
	MAPK9	Mitogen-activated protein kinase 9	NW_004677772.1
	ADCY1	Adenylate cyclase 1	NW_004676635.1
	ST6GALNAC3	ST6 N-acetylgalactosaminide alpha-2,6-sialyltransferase 3	NW_004676388.1
	ST8SIA5	ST8 Alpha-N-acetyl-neuraminide alpha-2,8-sialyltransferase 5	NW_004676540.1
	NPY1R	Neuropeptide Y receptor Y1	NW_004676768.1
	NPY	Neuropeptide Y	NW_004677092.1
	EGFR	Epidermal growth factor receptor	NW_004676665.1
	RPS6KA3	Ribosomal protein S6 kinase A3	NW_004676419.1
	CDH18	Cadherin-18	NW_004677258.1
	RPS23	Ribosomal protein S6 kinase A3	NW_004676419.1
	LOC101796003	Unknown	
	MBOAT2	Membrane bound O-acyltransferase domain containing 2	NW_004676614.1
	Novel00579	Unknown	
	Novel03624	Unknown	
	PRDM12	PR/SET domain 12	NW_004676566.1
	PENK	Proenkephalin	NW_004676497.1
	ERBB4	Erb-b2 receptor tyrosine kinase 4	NW_004677206.1
Victim group 31	VEGFD	Vascular endothelial growth factor D	NW_004676689.1
	PTGDS	Prostaglandin D2 synthase	NC_000009.11
	S100B	S100 calcium binding protein B	NW_004676788.1
	MST1	Macrophage stimulating 1	NW_004677459.1
	ADCY1	Adenylate cyclase 1	NW_004676635.1
	CRTC1	CREB regulated transcription coactivator 1	NW_004678889.1
	BMPR2	Bone morphogenetic protein receptor type 2	NW_004676788.1
	ATP8A1	ATPase phospholipid transporting 8A1	NW_004676708.1
	SLC24A2	Solute carrier family 24 member 2	NW_004676570.1
	PREX2	Phosphatidylinositol-3,4,5-tRisphosphate dependent Rac exchange factor 2	NW_004676991.1
	TMOD2	Tropomodulin-2	NW_004680033.1
	THRB	Thyroid hormone receptor beta	NC_040047.1
	ATP2B2	ATPase plasma membrane Ca2+ transporting 2	NW_004677729.1
	MAPK9	Mitogen-activated protein kinase 9	NW_004677772.1
	SORCS3	Sortilin related VPS10 domain containing receptor 3	NW_004676303.1
	APBA1	Abeta precursor protein binding family A member 1	NW_004676833.1
	KLHL1	Kelch like family member 1	NW_004676335.1
	BRAF	B-Raf proto-oncogene, serine/threonine kinase	NW_004677175.1
	USP46	Ubiquitin specific peptidase 46	NW_004676593.1
	BTBD9	BTB domain containing 9	NW_004677426.1
	KCNA1	Potassium voltage-gated channel subfamily A member 1	NW_004676532.1
	ERBB4	Brb-b2 receptor tyrosine kinase 4	NW_004677206.1
	RAB11FIP2	RAB11 family interacting protein 2	NW_004677141.1
	Novel02061	Unknown	
	NPY1R	Neuropeptide Y receptor Y1	NW_004676768.1
	ACAP3	ArfGAP with coiled-coil, ankyrin repeat and PH domains 3	NW_004676297.1
	IQSEC3	IQ motif and Sec7 domain 3	NW_004676462.1
	IQSEC1	IQ motif and Sec7 domain 1	NW_004676455.1
	ASAP1	ArfGAP with SH3 domain, ankyrin repeat and PH domain 1	NW_004676398.1
	Novel01018	Unknown	
	RAB4A	Member RAS oncogene family	NW_004677068.1
	PRDM12	PR/SET domain 12	NW_004676566.1

## Data Availability

All data and materials that support the conclusions of this article are fully described and available within the main text of the article. The key experimental data, including qRT-PCR validation results and behavioral observation statistics, are presented in the respective tables to ensure the reproducibility of the study’s core findings. For further detailed information regarding the experimental protocols, raw data records, and additional methodological details presented herein, interested parties may direct inquiries to the corresponding author (Shenglin Yang; E-mail: shenglinyang@126.com).
